# Dupilumab therapy following JAK inhibitor withdrawal in moderate–severe atopic dermatitis

**DOI:** 10.1111/dth.15750

**Published:** 2022-08-08

**Authors:** Lisa Kiely, Cathal O'Connor, Michelle Murphy

**Affiliations:** ^1^ Department of Dermatology South Infirmary Victoria University Hospital Cork Ireland; ^2^ Department of Medicine University College Cork Ireland

Dear Editor,

The advent of janus kinase inhibitors (JAKi) and targeted biologics is revolutionizing the treatment of recalcitrant atopic dermatitis (AD).^1^ JAKi have been shown to impair IL‐4‐ and IL‐13‐dependent differentiation of T_H_2 cells, improve skin barrier function, and suppress itch.^2^ Dupilumab inhibits IL‐4 and IL‐13 signaling via their shared IL‐4α subunit, suppressing the release of proinflammatory cytokines, chemokines, and immunoglobulin E.^3^ However, little is known about response to biologic therapy following JAKi failure or withdrawal.^4^ We present three patients who had treatment with upadacitinib/tofacitinib withdrawn but achieved disease remission with dupilumab.

Case 1 is a 27‐year‐old male with severe generalized AD who started treatment with upadacitinib in April 2019 following failure of potent topical corticosteroids (TCS) and methotrexate. He had one episode of herpes zoster while on upadacitinib. Despite initial improvement he subsequently developed a suberythrodermic flare of AD in November 2019 (EASI 42, DLQI 19). Therapy was switched to dupilumab in November 2019. Remission was achieved after 8 weeks (IGA 0) and has been maintained. Dupilumab has been well tolerated apart from conjunctivitis and facial dermatitis at treatment initiation, which has been treated with oral itraconazole and topical miconazole.

Case 2 is a 28‐year‐old male with severe generalized AD who started upadacitinib in September 2019 following failure of potent TCS, phototherapy, and methotrexate. He had moderate improvement in AD severity but developed severe lymphopenia (lowest count 0.5) during treatment. Upadacitinib was withdrawn in October 2021. Following discontinuation his AD deteriorated (EASI 25.4, DLQI 12). Therapy was switched to dupilumab in January 2022. Within 6 weeks of treatment his skin dramatically improved (EASI 2.6, DLQI 0) and his lymphopenia resolved. He remains in remission (IGA 0).

Case 3 is a 65 year‐old‐female with severe generalized AD who started upadacitinib in May 2019 following failure of potent TCS and methotrexate. She had a dramatic improvement but was diagnosed with a melanoma in situ in September 2019. Upadacitinib was discontinued, resulting in a flare of her AD. Therapy with tofacitinib was initiated in May 2020, which was discontinued in November 2021 due to lack of response (EASI 28.9, DLQI 20). Therapy was switched to dupilumab in November 2021, with significant improvement after 8 weeks (EASI 3.5, DLQI 4). She remains in remission (IGA 0).

Dupilumab was granted approval by the Food and Drug Administration (FDA) in 2017 and the European Medicines Agency (EMA) in 2019. Upadacitinib, a novel selective JAK1 inhibitor,^5^ was approved by the FDA in 2022 and EMA in 2021. Both treatments are licensed for patients with severe AD who failed to respond or are intolerant of conventional systemic treatment. Both have been shown to significantly improve AD severity and pruritus.^6^ Heads Up, a phase 3b trial, compared the safety and efficacy of dupilumab and upadacitinib. The trial demonstrated superior and more rapid benefit for upadacitinib over dupilumab in moderate to severe AD.^6^ Rates of serious infection, eczema herpeticum, herpes zoster, and laboratory‐related adverse events were higher for patients who received upadacitinib, whereas rates of conjunctivitis and injection‐site reactions were higher for patients who received dupilumab. Advantages of JAKi over monoclonal antibodies include oral dosing, more rapid onset of effect, and potentially better itch and disease control, although the safety profile of JAKi may be less desirable than targeted biologics.

We report three patients with severe AD who discontinued treatment with JAKi due to failure or intolerance, and were switched to dupilumab. All patients were clear or almost clear (IGA 0/1) within 8 weeks and had notable reductions in EASI assessments. This case series supports the use of dupilumab in patients with moderate–severe AD who require withdrawal of JAKi therapy. Expert guidelines are required to support clinicians in transitioning between these novel agents for AD.

## AUTHOR CONTRIBUTIONS

Cathal O'Connor and Michelle Murphy conceived of the research idea. Lisa Kiely collected data. Lisa Kiely and Cathal O'Connor wrote the paper and Lisa Kiely, Cathal O'Connor, and Michelle Murphy reviewed the paper.

## INFORMATION CONSENT

The patients in this case series provided written informed consent for the publication of their case details.

## Data Availability

Data available on request due to privacy/ethical restrictions.
